# Early Second-Trimester Serum MiRNA Profiling Predicts Gestational Diabetes Mellitus

**DOI:** 10.1371/journal.pone.0023925

**Published:** 2011-08-24

**Authors:** Chun Zhao, Jing Dong, Tao Jiang, Zhonghua Shi, Bin Yu, Yunlong Zhu, Daozhen Chen, Junrong Xu, Ran Huo, Juncheng Dai, Yankai Xia, Shiyang Pan, Zhibin Hu, Jiahao Sha

**Affiliations:** 1 Laboratory of Reproductive Medicine, Nanjing Medical University, Nanjing, China; 2 Department of Laboratory Medicine, The First Affiliated Hospital of Nanjing Medical University, Nanjing, China; 3 Department of Epidemiology and Biostatistics, Ministry of Education Key Laboratory of Modern Toxicology, School of Public Health, Nanjing Medical University, Nanjing, China; 4 Nanjing Maternity and Child Health Hospital of Nanjing Medical University, Nanjing, China; 5 Changzhou Maternity and Child Health Hospital, Changzhou, China; 6 Wuxi Maternity and Child Health Hospital, Wuxi, China; 7 Department of Histology and Embryology, Nanjing Medical University, Nanjing, China; 8 Institute of Toxicology, School of Public Health, Nanjing Medical University, Nanjing, China; National Institutes of Health - National Institute of Child Health and Human Development, United States of America

## Abstract

**Background:**

Gestational diabetes mellitus (GDM) is one type of diabetes that presents during pregnancy and significantly increases the risk of a number of adverse consequences for the fetus and mother. The microRNAs (miRNA) have recently been demonstrated to abundantly and stably exist in serum and to be potentially disease-specific. However, no reported study investigates the associations between serum miRNA and GDM.

**Methodology/Principal Findings:**

We systematically used the TaqMan Low Density Array followed by individual quantitative reverse transcription polymerase chain reaction assays to screen miRNAs in serum collected at 16–19 gestational weeks. The expression levels of three miRNAs (miR-132, miR-29a and miR-222) were significantly decreased in GDM women with respect to the controls in similar gestational weeks in our discovery evaluation and internal validation, and two miRNAs (miR-29a and miR-222) were also consistently validated in two-centric external validation sample sets. In addition, the knockdown of miR-29a could increase *Insulin-induced gene 1* (*Insig1*) expression level and subsequently the level of *Phosphoenolpyruvate Carboxy Kinase2* (*PCK2*) in HepG2 cell lines.

**Conclusions/Significance:**

Serum miRNAs are differentially expressed between GDM women and controls and could be candidate biomarkers for predicting GDM. The utility of miR-29a, miR-222 and miR-132 as serum-based non-invasive biomarkers warrants further evaluation and optimization.

## Introduction

Gestational diabetes mellitus (GDM), defined as any degree of glucose intolerance at first recognition during pregnancy, is one of the most common pregnancy complications and affects approximately 3–8% of all pregnancies [1], [2]. Importantly, the incidence is increasing with the increased prevalence of obesity among women at reproductive age [3], [4], [5], [6]. Although the detailed mechanism how the GDM happened remains poorly known, the GDM could lead to various adverse outcomes on pregnant women and their offspring, such as gestational hypertension, cesarean delivery, preterm birth, macrosomia and hyperbilirubinemia, as well as the predispositions to the development of metabolic syndrome and type 2 diabetes [7].

GDM is usually diagnosed at the end of the second trimester or early third trimester based on pregnancy physiology. As recommended by the American College of Obstetricians and Gynecologists (ACOG) and the American Diabetes Association (ADA), the serum-based screening for GDM typically begins at 24–28 weeks of gestation; however, as many use a 2-step process, testing may not be completed until 32 weeks [8]. This leaves little time for intervention and management of GDM. Detection of women at higher risk of GDM early in pregnancy is a desirable goal, because interventions, such as diet, medication and exercise, may be applied earlier to have a positive effect on maternal and fetal outcomes.

MiRNAs are a class of small non-coding RNAs that function as translational repressors involved in many important biological processes [9], [10]. Specifically, miRNAs are required for pancreatic development and the regulation of glucose stimulated insulin secretion [11], [12], [13]. Growing evidence indicates that miRNAs are involved in the pathogenesis of diabetes and that a number of miRNAs have been reported to be differently expressed in pancreatic β-cells, liver, adipose tissue, and/or skeletal muscle of animal models of type 1 or type 2 diabetes, such as miR-146a, miR-21, miR-29a, miR-34a, miR-222, and miR-375 [14]. Recently, miRNAs were found to be abundant in human serum, and the serum miRNAs attracted most attention because of their unique merits (i.e., stable, easily to be detected, and potentially disease-specific) [15]. Although the exact mechanism how miRNAs enter into the serum and whether they are biologically functional or simply biomarkers for unknown etiological factors is still a big obsession, a recent study reported that miRNAs could be selectively packaged into microvesicles and actively secreted [16]. Therefore, serum miRNAs could be a potential independent predictive system for different diseases, compared with biomarkers derived from target tissues.

In this study, we hypothesized that serum miRNA could serve as candidate biomarkers for predicting GDM in the relatively early pregnancy. To address this hypothesis, we systematically screened serum miRNAs by using the Taqman Low Density Array (TLDA) chips followed by individual quantitative reverse transcriptase polymerase chain reaction (qRT-PCR) assays. We also performed internal and two-centric external validations by using individual qRT-PCR assays. Finally, *in vitro* analysis of miR-29a was conducted in HepG2 cell lines to investigate the potential role of miR-29a in GDM development.

## Results

The characteristics of participants are summarized in Table 1. The cases and controls were well matched on age, BMI, gestational week and gravidity. For the discovery and internal validation samples, serum glucose levels were slightly higher for GDM patients, and in all samples, only eight subjects have a serum glucose level >7.8 mmol/L but none of them >11.0 mmol/L at that time.

**Table 1:** Characteristics of the study population.

Variable	Discovery Stage		*P* a	Internal Validation		*P* a	External Validation (Wuxi)		*P* a	External Validation (Changzhou)		*P* a
	case	Control		case	control		case	control		case	control	
Age(years)	28.79±2.21	29.46±1.89	0.267	29.21±1.61	28.57±1.80	0.074	26.88±1.96	26.88±2.06	1	27.94±2.72	26.75±1.65	0.146
BMI(kg^2^/m)	21.44±1.70	21.9±1.81	0.374	21.50±1.85	21.31±2.17	0.638	20.62±1.25	20.73±1.37	0.811	21.95±1.99	22.13±1.93	0.794
Gestational Week	17.40±0.70	17.16±0.79	0.262	17.09±0.78	17.23±0.76	0.373	17.55±0.92	17.53±0.93	0.935	16.95±0.85	17.17±0.88	0.470
Serum glucose	5.30±0.90	4.76±0.90	0.042	5.52±1.06	4.96±1.20	0.041	5.52±2.09	5.35±1.31	0.772	5.31±1.99	5.23±0.48	0.875
OGTT												
2+	17			24			14			8		
3+	7			11			2			4		
4+	0			1			0			4		
Gravidity												
1	14	12	0.925^b^	20	20	0.351^b^	10	10	0.470^b^	11	11	1.000^b^
2	7	8		8	13		4	6		4	5	
> = 3	3	4		8	3		2	0		1	0	

a Student's t test.

b Fisher's exact test.

**Figure 1:**
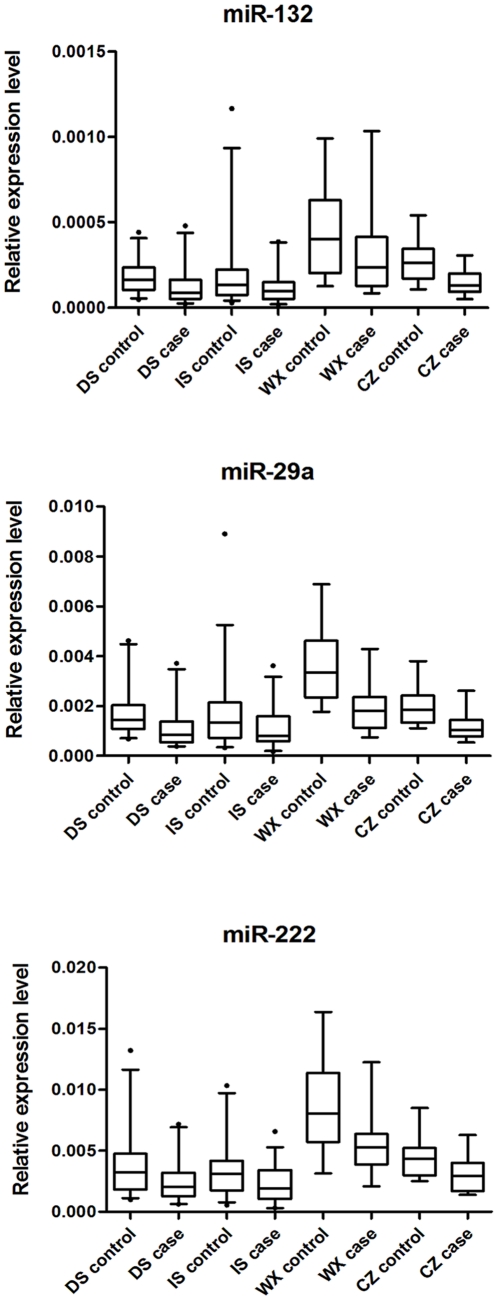
Expression levels of miRNAs. DS: discovery stage; IS: internal validation stage; WX: Wuxi; CZ: Changzhou Three serum miRNAs were quantified by qRT-PCR in patients with subsequent GDM and matched controls (n = 24, 36, 16 and 16 each for DS, IS, WX and CZ, respectively). The Box-whisker Plot represented the relative expression levels of miRNAs that were determined by the equation 2−ΔCT, in which ΔCT = CT sample-CT cel-39. The bottom and top of the box were the 5th and 95th percentiles, and the band near the middle of the box was the 50th percentile of the relative expression levels of miRNAs. Any data beyond these whiskers were shown as points.

In all subjects, 73 miRNAs showed ΔΔC_T_>3 (i.e. 8-fold) by the pooled TLDA chip assay (Table S1). Based on both scientific and applicable considerations, we selected miRNAs that had at most 35 of C_T_ value by TLDA in both two pools for further individual qRT-PCR confirmation. As a result, 10 miRNAs were identified and subjected to individual qRT-PCR analyses on 48 discovery-stage samples (Table 2). Because the expression pattern of miR-222 was reported to be associated with hyperglycemia, we included it in further analysis, although it did not meet the criteria (TLDA: ΔΔC_T_ = 2.834) [17]. In the discovery stage, the expression levels of three miRNAs (i.e., miR-132, miR-29a, and miR-222) were significantly different between GDM women and controls (*P* = 0.042, 0.032, and 0.041 for miR-132, miR-29a, and miR-222, respectively) (Figure 1).

**Table 2:** Results of 11 miRNAs in the discovery stage.

miRNA	GDM					No GDM					ΔΔCT^b^	*P* c
	N	ΔCT^a^	mean	SD	median	N	ΔCT^a^	mean	SD	median		
hsa-miR-1	24	6.336	4.03*10-5	3.59*10-5	2.98*10-5	24	2.781	4.92*10-5	4.67*10-5	3.37*10-5	3.894	0.463
hsa-miR-125b	24	9.248	5.95*10-5	3.25*10-5	5.55*10-5	24	4.73	7.73*10-5	3.75*10-5	7.07*10-5	4.857	0.085
**hsa-miR-132**	24	7.27	11.98*10-5	10.47*10-5	8.75*10-5	24	3.77	17.88*10-5	9.00*10-5	16.24*10-5	3.839	0.042
**hsa-miR-29a**	24	7.325	11.20*10-4	8.18*10-4	8.33*10-4	24	3.766	17.01*10-4	9.89*10-4	14.38*10-4	3.898	0.032
hsa-miR-203	24	8.197	1.80*10-5	1.15*10-5	1.44*10-5	24	4.757	2.55*10-5	1.58*10-5	2.10*10-5	3.779	0.065
**hsa-miR-222**	24	3.203	2.43*10-3	1.72*10-3	2.02*10-3	24	0.708	3.76*10-3	2.57*10-3	3.26*10-3	2.834	0.041
hsa-miR-378	24	8.353	10.07*10-4	5.77*10-4	9.00*10-4	24	5.214	11.17*10-4	6.86*10-4	9.82*10-4	3.478	0.550
hsa-miR-518d-3p	24	7.26	8.22*10-6	9.79*10-6	4.43*10-6	24	3.814	8.68*10-6	8.01*10-6	5.49*10-6	3.785	0.860
hsa-miR-632	24	7.348	1.68*10-4	1.05*10-4	1.44*10-4	24	4.205	2.07*10-4	1.12*10-4	1.80*10-4	3.482	0.226
hsa-miR-923	24	3.324	2.70*10-3	2.84*10-3	1.62*10-3	24	0.077	3.48*10-3	6.25*10-3	1.45*10-3	3.586	0.584
hsa-miR-99a	24	9.257	7.07*10-5	3.40*10-5	6.03*10-5	24	5.785	8.88*10-5	4.66*10-5	8.44*10-5	3.811	0.131

a TLDA results of 24 pooled samples, ΔCT = C_Tsample_−C_TRNU6B_.

b ΔΔC_T_ = ΔC_Tcase_−ΔC_Tcontrol_−ΔC_TCel-39_ from TLDA data.

c Student's *t* test from individual assay data.

As shown in Table 3, the expression levels of the three miRNAs were significantly different between cases and controls in internal validation-stage samples (*P* = 0.034, 0.045, and 0.016 for miR-132, miR-29a, and miR-222, respectively), while miR-29a and miR-222 were still significantly differently expressed in the two-centric external validation-stage samples (Wuxi: *P* = 0.001 and 0.017 for miR-29a and miR-222, respectively; Changzhou: *P* = 0.001 and 0.019 for miR-29a and miR-222, respectively) (Table 3 and Figure 1).

**Table 3:** Expression of the identified three miRNAs in the validation stages.

Validation stage	miRNAs	GDM				No GDM				*P* a
		N	Mean	SD	Median	N	Mean	SD	Median	
Internal validation	mir-132	36	12.15*10-5	9.36*10-5	9.85*10-5	36	21.24*10-5	23.41*10-5	13.34*10-5	0.034
	mir-29a	36	11.11*10-4	8.02*10-4	7.89*10-4	36	17.06*10-4	15.54*10-4	13.67*10-4	0.045
	mir-222	36	2.32*10-3	1.54*10-3	1.90*10-3	36	3.51*10-3	2.45*10-3	2.97*10-3	0.016
External validation	mir-132	16	3.23*10-4	2.56*10-4	2.35*10-4	16	4.32*10-4	2.56*10-4	4.00*10-4	0.235
Wuxi	mir-29a	16	19.66*10-4	9.69*10-4	17.97*10-4	16	36.74*10-4	15.85*10-4	33.35*10-4	0.001
	mir-222	16	5.72*10-3	2.71*10-3	5.27*10-3	16	8.73*10-3	3.91*10-3	8.02*10-3	0.017
External validation	mir-132	16	14.62*10-5	7.71*10-5	13.02*10-5	16	27.55*10-5	11.75*10-5	26.27*10-5	0.001
Changzhou	mir-29a	16	11.39*10-4	5.18*10-4	10.31*10-4	16	19.61*10-4	7.16*10-4	18.44*10-4	0.001
	mir-222	16	3.10*10-3	1.45*10-3	2.94*10-3	16	4.48*10-3	1.69*10-3	4.36*10-3	0.019

Expression levels were relative to C_TCel-39_.

a Student's *t* test.

We further plotted the Receiver Operating Characteristic (ROC) curves and calculated the Area under the ROC Curve (AUC) to assess the sensitivity and specificity of the miRNA signature individually and in combination for GDM predicting. In the consideration of the heterogeneity of different centers, the AUC analysis was only performed in the samples from the discovery and internal validation stages (from Nanjing Maternity and Child Health Hospital of Nanjing Medical University). As a result, for single miRNA, the AUC was 64.2%, 65.8% and 60.0% for miR-132, miR-29a and miR-222, respectively; and the AUC was 66.9% (sensitivity = 66.7% and specificity = 63.3%), when we combined all three miRNAs. Overall, 22 (18.3%) false-positive and 20 (16.7%) false-negative subjects were found in the 120 samples (detailed data not shown).

**Figure 2:**
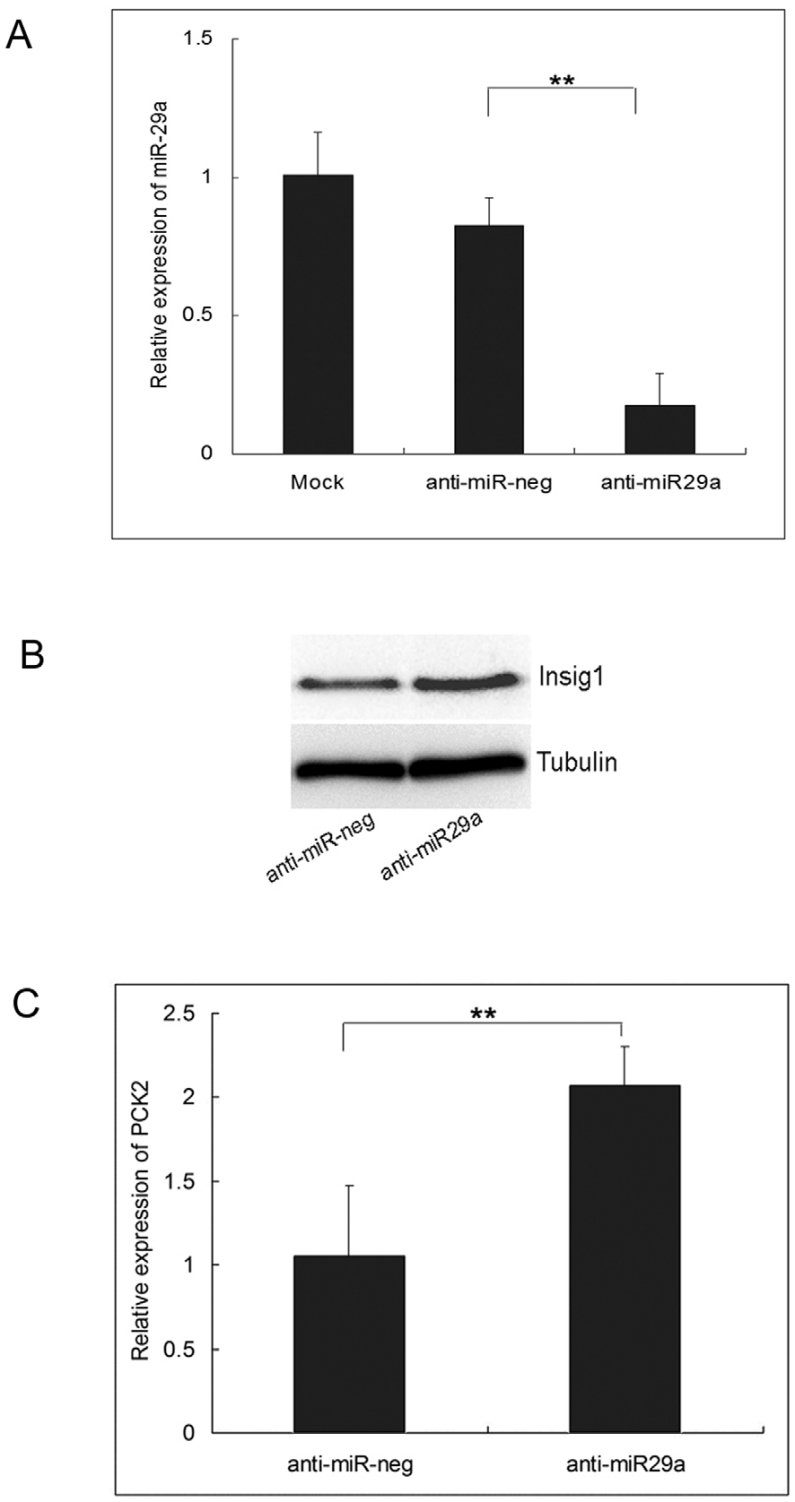
A. Expression of miR-29a in HepG2 cells transfected with 100 nM anti-miR-29a, anti-miR-neg or mock to determine the knockdown efficiency of anti-miR-29a. ** *P*<0.01. B. Western blots for Insig1 or tubulin using protein extracts from HepG2 cells transfected with 100 nM anti-miR-29a or anti-miR-neg. C. Expression of PCK2 in HepG2 cells transfected with 100 nM anti-miR-29a, anti-miR-neg to determine the regulation of miR-29a on the expression of PCK2. Data represented three independent experiments ±S.E.M. with n = 3. ** *P*<0.01.

Because insulin-induced gene 1 (*Insig1*) was demonstrated as a target gene of miR-29a, and Krapivner *et al.* showed that *Insig1* played a role in glucose homeostasis and that the action of *Insig1* was related to sterol regulatory element-binding proteins (SREBP)-mediated regulation of the hosphoenolpyruvate Carboxy Kinase 2 (*PCK2*), a key enzyme in gluconeogenesis and glycolysis, we further investigated the relationship between *Insig1* levels and miR-29a expression, and subsequently altered expression of *PCK2* [18], [19]. As shown in Figure 2, the expression of miR-29a in HepG2 cells was significantly decreased when transfected with anti-miR-29a compared with that in those cells transfected with anti-miR-neg. In addition, the expression of Insig1 protein in HepG2 cells was increased when miR-29a was down-regulated (*P* < 0.01), and cells with down-regulated miR-29a also had a significantly higher level of *PCK2* mRNA expression (*P* < 0.01).

## Discussion

Numerous studies have demonstrated the associations between GDM and both neonatal morbidity and maternal complications. Most protocols for screening and diagnosis are initiated at 24–28 gestational weeks, and previously studies have demonstrated a number of serum markers (e.g. sex hormone-binding globulin, the homeostasis model assessment index and C-reactive protein) measured in the first and early second trimesters are associated with the later diagnosis of GDM [20], [21], [22], [23]. The present study firstly revealed a serum miRNA signature for predicting GDM in the early second trimester and found that signatures of the miRNAs (mir-29a and mir-222 and miR-132) aberrant expressed prior to serum glucose abnormality.

Several studies had reported that human serum/plasma could serve as a class of novel promising noninvasive biomarkers for diseases [15], [24], [25], [26], [27]. Previously, we had used a two-stage study to investigate the role of serum miRNAs in predicting prognosis of nonsmall-cell lung cancer (NSCLC) and found that expression levels of four miRNAs (miR-486, miR-30d, miR-1 and miR-499) were significantly associated with NSCLC survival [24]. Ng *et al.* found that the expression levels of plasma miR-92 were significantly elevated in colorectal cancer patients and well distinguished from gastric cancer, inflammatory bowel disease and normal subjects [25]. Fichtlscherer *et al.* reported that the expression levels of miR-126, miR-17, miR-92a and miR-155 were significantly reduced in the plasma of coronary artery disease patients compared with healthy controls' [26]. Finally, plasma miR-1 expression level was found to be significantly higher in acute myocardial infarction (AMI) patients compared with non-AMI subjects, and the level was dropped to normal on discharge following medication [27].

For diabetes, Chen *et al.* compared the serum miRNA expression in type 2 diabetic patients with that in healthy individuals and found that the serum miRNA profiling was significantly different between patients and controls [15]. Recently, Zampetaki *et al.* used the microarray screening and qRT-PCR methods to assess the plasma miRNA profiling in type 2 diabetes and found that expression levels of miR-20b, miR-21, miR-24, miR-15a, miR-126, miR-191, miR-197, miR-223, miR-320, and miR-486 were lower in prevalent type 2 diabetes [28]. Kong *et al.* found that serum miR-29a was significantly down-regulated in type 2 diabetes susceptible individuals with normal glucose tolerance (s-NGT) and in pre-diabetes individuals compared with type 2 diabetes patients (n-T2D), but no significantly different expression between s-NGT and pre-diabetes individuals was observed [29]. The differences between our study and those two previous studies are: (1) Compared to non-pregnant women, pregnant women were typically characterized as a “diabetogenic state” because of the placental hormones. The human placenta is considered an active organ playing a role in the aggravated insulin resistance by secreting substances such as inflammatory cytokines and adipokine [30], [31], [32]. (2) We tested serum miRNAs in the early second trimester when the subjects were not having diagnostic GDM, and none of them have increased blood glucose >11.0 mmol/L.

As of today, we just begin to understand the complex mechanisms that culminate into the GDM phenotype and its complications. *Insig1* was a validated target gene of miR-29a and a blocker of proteolytic activation of *SREBPs*, which activated genes regulating cholesterol and fatty acid metabolism and possibly genes involved in glucose homeostasis because of the target gene of *SREBPs* was *PCK2*, a key enzyme in gluconeogenesis in hepatic cells [18], [33], [34], [35], [36], [37], [38], [39]. Therefore, overexpression of miR-29a causing decreased levels of Insig1 may lead to a reduced expression of *PCK2*, exhibiting reduced glucose concentration [19]. A recent study reported that high glucose could reduce the levels of miR-29a in HK-2 cells [40]. In our study, we found that the knockdown of miR-29a could increase Insig1 expression level and subsequently increased the level of *PCK2*, which may lead to elevation of the glucose level, and the serum miR-29a expression decreased ahead of the elevation of serum glucose. Taken together, we inferred that miR-29a is a negative regulator of serum glucose. Mir-222 is located on Xp11.3, and the up-regulation of miR-222 may be involved in cell cycle regulation through its control of cyclin-dependent kinase inhibitor (p27^Kip1^) expression [41]. It has been shown that miR-132 was reported significantly differently expressed in the pancreatic islets of the GK rats, and the up-regulated miR-132 expression could decrease production of key proteins of the insulin exocytotic machinery and reduce insulin secretion of the GK rats [42]. However, the biological function of miR-222 and miR-132 in GDM was not clear up to date. Additional investigation of the regulatory mechanism of these miRNAs and their target mRNAs may improve our understanding of the molecular pathogenesis of GDM as well as the effectiveness in identifying potential therapeutic targets and surveillance markers for GDM.

Major strengths of our study include the use of a multistage study design. We collected blood samples of pregnant women at their 16–19 gestational weeks, and GDM was diagnosed at 25–28 gestational weeks. It shed light on the possible effects of earlier intervention and greater aggressive treatment on maternal and fetal outcomes. Furthermore, we spiked-in cel-mir-39 to normalize the results and conducted qRT-PCR with equal numbers of cases and control on the same plate together with cel-mir-39. However, some limitations also need to be addressed. First, the sample size of the external validation was relatively small, which may present underpowered results. Second, the expression levels of miRNAs were slightly higher in external validation samples than in discovery and internal validation samples that may be the consequence of lower stored temperature, which lead to less degradation of miRNAs. Therefore, we cannot combine external validation results when performing predictive analyses. Third, because the investigation of the role of miR-29a in insulin signaling was just a replication of previous studies [18], [19], additional functional studies are needed to further investigate the role miR-29a as well as miR-222 and miR-132 in GDM. Finally, although we demonstrated that the serum miRNAs (miR-29a, miR222 and miR-132) were differentially expressed between GDM women and controls, the clinical application of these miRNAs in predicting GDM still needs further investigation and optimization.

## Materials and Methods

### Study Design and Study Population

We designed a multistage retrospective nested case-control study to determine whether serum miRNA profiling could predict GDM development and whether the predicting property may be prior to the change of blood glucose. All pregnant women provided blood samples, when they received prenatal care at 16–19 gestational weeks. The sera were isolated within 4 hours after collection and tested for levels of random glucose using Olympus AU5400 analyzer (Olympus Diagnostic Systerms, Southall, Middlesex, UK). The sera of discover and internal validation samples were stored at −20°C, and those of external validation were stored at −70°C. At 24–28 weeks of gestation, all participants had a subsequent 50-g glucose challenge test (GCT), and women with an abnormal 1-h post-GCT glucose level (≥7.8 mmol/L) would undergo 3-h 75-g oral glucose tolerance test (OGTT), assessing the blood glucose levels at 0, 1, 2 and 3 h after glucose administration. Those who had results ≥2 abnormal values on OGTT in accordance with ADA guidelines were defined as cases; while those who passed GCT and/or OGTT were chosen as controls. To control sample heterogeneity, only those controls matched with cases on age, body mass index (BMI) and pregnant weeks at the time of blood collecting were included in this study. We excluded subjects with diabetes history, multiple gestation, other pregnancy complications, and those found to have glucose intolerance at <20 weeks of gestation, and BMI >26, age >33 years, and small-for gestational-age infants.

To detect the generalizable signatures of miRNAs for the prediction of GDM, we pooled serum samples of 24 cases (from Nanjing Maternity and Child Health Hospital of Nanjing Medical University, Nanjing) and 24 controls, respectively, to subject them to TLDA chip screening in the discovery stage. Then, we performed individual qRT-PCR for the discovery-stage samples to further filter signals of the screened miRNAs due to heterogeneity in the subjects, as described previously [24]. Subsequently, a two-stage validation, including an internal validation and a two- centric external validations, was conducted to confirm the results from the discovery stage. For internal validation, 36 cases (again from Nanjing Maternity and Child Health Hospital of Nanjing Medical University, Nanjing) and 36 controls were tested. The controls used for discovery and internal validation stages were recruited at the same hospital and during the same time period as cases between July 2008 and June 2009. The external validation was conducted with samples from two independent centers of Wuxi and Changzhou, respectively. In brief, 16 cases and 16 controls from Wuxi Maternity and Child Health Hospital were recruited in parallel between March 2008 and December 2008, and 16 cases and 16 controls were recruited in parallel between July 2006 and June 2007 from Changzhou Maternity and Child Health Hospital. This study was approved by the institutional review boards of Nanjing Medical University and the participating hospitals, and a written informed consent was also obtained from each participant. Clinical information and birth outcomes were collected from the obstetric electronic medical records.

### Serum preparation and RNA extraction

Five-ml venous blood was collected from each participant using a procoagulant drying tube, when they received prenatal care at 16–19 weeks of gestation. The whole blood was separated into serum and cellular fractions by centrifugation at 4,000 rpm for 10 min, followed by 12000 rpm for 15 min to completely remove cell debris.

Isolation of serum total RNA was described previously with some modifications [43]. In brief, the Trizol Reagent (Invitrogen, Carlsbad, CA) was used for serum denaturizing and Qiagen miRNeasy Mini kit (Qiagen, Valencia, CA) for RNA collection and purification according to the manufacturer's protocol. Because there was no consensus on the use of housekeeping miRNA for the serum qRT-PCR analysis, after the initial denaturizing step, we routinely spiked in synthetic *C.elegans* miR-39 (cel-mir-39, 5′-ucaccggguguaaaucagcuug -3′) to a final concentration of 10^−4^ pmol/µl for all samples in order to control variations in RNA extraction and/or purification procedures because of the absence of homologous sequences in humans [44]. Furthermore, all study subjects were recruited during the same period, stored under the same conditions (for each center), and the samples were handled in equal volume in each experiment step to control the potential bias.

### TLDA chip assays and qRT-PCR

In the discovery stage, we used TLDA Chips (human microRNA panel V2.0, Applied Biosystems Inc, CA, USA) to screen differentially expressed miRNAs from the two pooled samples. A total of 960-µl serum from each pool (24 samples) was used. Megaplex RT reactions and pre-amplification reactions were run according to the manufacture's protoco, in which 75-µl 0.1× TE was added to PreAmp product, and 9 -µl diluted PreAmp product was used to run the RT-PCR reactions by dispensing 100 µl of the PCR reaction mix into each port of the TaqMan MicroRNA Array. The default PCR procedure was used, and the analysis was performed by using RQ manager software (Applied Biosystems Inc.). ΔC_T_ and ΔΔC_T_ were calculated using the following mathematical formula: ΔC_T_ = C_T sample_−C_T RNU6B_, ΔΔC_T_ = ΔC_T case_−ΔC_T control_. Finally, the ΔΔC_T_ was normalized against the cel-miR-39.

Then, we used TaqMan microRNA probes (Applied Biosystems Inc.) to perform qRT-PCR assays according to the manufacturer's instructions [45], [46]. The probe information was shown in Table S2. Equal volume of samples was used in each step from serum purification to qRT-PCR. The total RNA was reverse-transcribed to cDNA by using a TaqMan microRNA RT Kit and stem-loop RT primers (Applied Biosystems Inc.). RT-PCR was performed using the TaqMan PCR kit on the ABI 7900 Real-Time PCR System (Applied Biosystems Inc.). The reactions were initiated in a 384-well optical plate at 95°C for 5 min, followed by 40 cycles of 95°C for 15 s and 60°C for 1 min. We assigned equal number of patients and controls on one plate and run the RT-PCR for target miRNAs and cel-miR-39 simultaneously. All reactions, including no-template controls, were run in triplicate. The CT values were determined using the fixed threshold settings. The relative expression levels of target miRNAs were determined by the equation 2^−ΔCT^, in which ΔC_T_ = C_T sample_−C_T cel-39_.

### Cell Culture

The human liver carcinoma cell line HepG2 was purchased from the cell culture center of the Chinese Academy of Medical Sciences (Beijing, China). Cells were maintained in Dulbecco's modified Eagle's medium (DMEM) supplemented with 1 mg/ml glucose, 10% heat-inactivated FBS, 100 IU/ml penicillin and 100 mg/ml streptomycin (Gibco/Life Technologies, Paisley, UK) and incubated at 37°C in a humidified incubator with 5% CO_2_.

### Transient transfection of anti-miR miRNA inhibitors

HepG2 cells were seeded in 6-well plates at 10^5^ cells/well. After 24 h, 100 nM anti-hsa-miR-29a or anti-miR-neg (Gene-pharma) were transiently transfected into HepG2 cells by Lipofectamine 2000 (Invitrogen). Forty-eight hours after transfection, cells were extracted to perform western blot and real-time PCR.

### Quantitative RT-PCR

Quantitative RT-PCR was performed to determine the expression level of mRNAs of the hosphoenolpyruvate Carboxy Kinase2 (*PCK2*) gene. RNA from HepG2 cells was isolated with Trizol Reagent (Invitrogen, Carlsbad, CA, USA) according to the manufacturer's protocol. Real-time PCR was performed with Power SYBR Green PCR Master Mix (Applied Biosystems Inc.). Primers 5′-ACCCTGCGAGTGCTTAGTG-3′ and 5′-TTCTCAGCCTCAGTTCCATC-3′ were used to amplify the *PCK2* gene, and primers 5′-CAAGAGATGGCCACGGCTGCT-3′ and 5′-TCCTTCTGCATCCTGTCGGCA-3′ were used to amplify the β-actin gene to normalize the expression level of *PCK2*. All quantitative real-time PCR reactions were run by using the ABI7900 Real-Time PCR System (Applied Biosystems Inc.) and performed in triplicate. Relative expression of *PCK2* was calculated using the equation 2^−ΔCT^ in which ΔC_T_ = C_T PCK2_−C_T β-actin_.

### Western blot analysis

The insulin-induced gene1 (Insig1) protein was analyzed by western blot from the total cell lysate as described previously [47]. Proteins were extracted with the urea lysis buffer. Proteins (50 µg) were fractionated by electrophoresis on 12% SDS polyacrylamide gel and transferred onto a nitrocellulose membrane (GE Healthcare, San Francisco, CA). The membranes were blocked in Tris-buffered saline (TBS) containing 5% nonfat milk powder for 1 h and then incubated in polyclonal anti-Insig1 (1∶500, Abcam, Cambridge, MA, USA) and anti-β-tubulin (1∶2000, Abcam, Cambridge, MA) diluted in TBS/5% non-fat milk powder overnight. The expression of β-tubulin was used as the loading control. Membranes were washed three times (10 min each) with TBS and then incubated for 1 h with horseradish peroxidase (HRP)-conjugated goat anti-rabbit IgG (1∶1000; Beijing ZhongShan Biotechnology CO., Beijing). Specific proteins were detected using an ECL kit and AlphaImager (FluorChem 5500, Alpha Innotech, San Leandro, CA). The protein expression level was analyzed by AlphaEaseFC software (Alpha Innotech, San Leandro, CA).

### Statistical Analysis

Differences in demographic and clinical characteristics and mean expression levels of miRNAs were evaluated by χ^2^ tests or the student's *t* test between GDM women and controls. To investigate the effectiveness of the three-miRNA (miR-132, miR-29a and miR-222) signature for GDM predicting, a risk score analysis was constructed. The upper 25% reference interval of each miRNA value in controls of the discovery stage was set as the threshold to code the expression level of the corresponding miRNA for each sample as 0 and 1 in discovery and internal validation stages (from Nanjing Maternity and Child Health Hospital of Nanjing Medical University). The risk score of each miRNA was calculated using the weights by the regression coefficient that was derived from the univariate logistic regression analysis of each miRNA. We further assigned each patient a risk score function according to a linear combination of the expression level of the miRNAs. The risk score = (−1.2154×expression level of miR-132)+(−1.608385×expression level of miR-29a)+(−0.929536×expression level of miR-222). The AUC was calculated for each and the combination of the three miRNAs, respectively, in orders to assess the individual and combined effects of the miRNAs on GDM predicting. All the statistical analyses were performed with Stata version 9.2 (Stata Corporation, College Station, TX, USA). A *P* value of less than.05 was considered statistically significant, and all tests were two tailed.

## Supporting Information

Table S1 **List of miRNA in TLDA chips.**
(DOC)

Table S2 **AB assay ID of the miRNAs.**
(DOC)
