# Supplementary material for: Early Second-Trimester Serum MiRNA Profiling Predicts Gestational Diabetes Mellitus
Source: PLoS One. 2011 Aug 24;6(8):e23925. doi: 10.1371/journal.pone.0023925 (PMC3161072; doi:10.1371/journal.pone.0023925)
Supplement: Table S1 — List of miRNA in TLDA chips. (DOC) [file pone.0023925.s001.doc]

**Table S1. List of miRNA in TLDA chips**

| TLDA | miRNA | C_T case_ | C_T control_ | TLDA | miRNA | C_T case_ | C_T control_ |
| --- | --- | --- | --- | --- | --- | --- | --- |
| A | has-miR-155-4395459 | 40 | 40 | A | hsa-miR-127-3p-4373147 | 40 | 35.949 |
| A | hsa-let-7a-4373169 | 40 | 40 | A | hsa-miR-127-5p-4395340 | 40 | 40 |
| A | hsa-let-7b-4395446 | 29.02 | 31.102 | A | hsa-miR-128-4395327 | 33.977 | 40 |
| A | hsa-let-7c-4373167 | 37.001 | 40 | A | hsa-miR-129-3p-4373297 | 40 | 40 |
| A | hsa-let-7d-4395394 | 30.945 | 33.007 | A | hsa-miR-129-5p-4373171 | 40 | 40 |
| A | hsa-let-7e-4395517 | 29.997 | 31.985 | A | hsa-miR-130a-4373145 | 33.963 | 40 |
| A | hsa-let-7f-4373164 | 40 | 40 | A | hsa-miR-130b-4373144 | 40 | 40 |
| A | hsa-let-7g-4395393 | 30.983 | 32.954 | A | hsa-miR-132-4373143 | 33.004 | 31.983 |
| A | hsa-miR-100-4373160 | 33.952 | 33.968 | A | hsa-miR-133a-4395357 | 32.918 | 34.014 |
| A | hsa-miR-101-4395364 | 36.928 | 40 | A | hsa-miR-133b-4395358 | 40 | 40 |
| A | hsa-miR-103-4373158 | 32.972 | 40 | A | hsa-miR-134-4373299 | 33.963 | 33.972 |
| A | hsa-miR-105-4395278 | 40 | 40 | A | hsa-miR-135a-4373140 | 40 | 40 |
| A | hsa-miR-106a-4395280 | 26.946 | 27.935 | A | hsa-miR-135b-4395372 | 40 | 40 |
| A | hsa-miR-106b-4373155 | 31.945 | 31.957 | A | hsa-miR-136-4373173 | 40 | 40 |
| A | hsa-miR-107-4373154 | 40 | 40 | A | hsa-miR-137-4373301 | 40 | 40 |
| A | hsa-miR-10a-4373153 | 34.97 | 40 | A | hsa-miR-138-4395395 | 40 | 40 |
| A | hsa-miR-10b-4395329 | 40 | 40 | A | hsa-miR-139-3p-4395424 | 40 | 40 |
| A | hsa-miR-122-4395356 | 29.972 | 30.995 | A | hsa-miR-139-5p-4395400 | 29.966 | 30.967 |
| A | hsa-miR-124-4373295 | 40 | 40 | A | hsa-miR-140-3p-4395345 | 33.953 | 34.996 |
| A | hsa-miR-125a-3p-4395310 | 40 | 40 | A | hsa-miR-140-5p-4373374 | 32.979 | 32.945 |
| A | hsa-miR-125a-5p-4395309 | 33.946 | 40 | A | hsa-miR-141-4373137 | 40 | 35.992 |
| A | hsa-miR-125b-4373148 | 34.982 | 32.943 | A | hsa-miR-142-3p-4373136 | 30.967 | 32.97 |
| A | hsa-miR-126-4395339 | 26.964 | 26.949 | A | hsa-miR-142-5p-4395359 | 35.975 | 35.953 |
| A | hsa-miR-143-4395360 | 35.96 | 40 | A | hsa-miR-185-4395382 | 32.007 | 33.013 |
| A | hsa-miR-1-4395333 | 32.07 | 30.994 | A | hsa-miR-186-4395396 | 30.902 | 31.952 |
| A | hsa-miR-145-4395389 | 31.971 | 35.022 | A | hsa-miR-187-4373307 | 40 | 40 |
| A | hsa-miR-146a-4373132 | 26.954 | 26.939 | A | hsa-miR-188-3p-4395217 | 40 | 40 |
| A | hsa-miR-146b-3p-4395472 | 40 | 40 | A | hsa-miR-18a-4395533 | 31.975 | 40 |
| A | hsa-miR-146b-5p-4373178 | 29.965 | 31.967 | A | hsa-miR-18b-4395328 | 40 | 40 |
| A | hsa-miR-147-4373131 | 40 | 40 | A | hsa-miR-190-4373110 | 40 | 40 |
| A | hsa-miR-147b-4395373 | 40 | 40 | A | hsa-miR-191-4395410 | 25.933 | 27.956 |
| A | hsa-miR-148a-4373130 | 32.896 | 34.989 | A | hsa-miR-192-4373108 | 32.958 | 32.946 |
| A | hsa-miR-148b-4373129 | 40 | 36.011 | A | hsa-miR-193a-3p-4395361 | 40 | 40 |
| A | hsa-miR-149-4395366 | 40 | 40 | A | hsa-miR-193a-5p-4395392 | 33.044 | 33.018 |
| A | hsa-miR-150-4373127 | 27.943 | 28.942 | A | hsa-miR-193b-4395478 | 31.965 | 34.046 |
| A | hsa-miR-152-4395170 | 34.943 | 34.932 | A | hsa-miR-194-4373106 | 34.993 | 35.971 |
| A | hsa-miR-153-4373305 | 40 | 40 | A | hsa-miR-195-4373105 | 31.953 | 32.983 |
| A | hsa-miR-154-4373270 | 40 | 40 | A | hsa-miR-196b-4395326 | 40 | 33.958 |
| A | hsa-miR-15a-4373123 | 40 | 40 | A | hsa-miR-197-4373102 | 28.972 | 30.916 |
| A | hsa-miR-15b-4373122 | 33.005 | 33.965 | A | hsa-miR-198-4395384 | 40 | 40 |
| A | hsa-miR-16-4373121 | 24.96 | 25.955 | A | hsa-miR-199a-3p-4395415 | 31.004 | 31.955 |
| A | hsa-miR-17-4395419 | 26.924 | 27.966 | A | hsa-miR-199a-5p-4373272 | 40 | 40 |
| A | hsa-miR-181a-4373117 | 32.953 | 32.949 | A | hsa-miR-199b-5p-4373100 | 40 | 40 |
| A | hsa-miR-181c-4373115 | 35.964 | 40 | A | hsa-miR-19a-4373099 | 29.983 | 29.927 |
| A | hsa-miR-182-4395445 | 35.955 | 35.963 | A | hsa-miR-19b-4373098 | 25.002 | 25.981 |
| A | hsa-miR-183-4395380 | 35.04 | 35.905 | A | hsa-miR-200a-4378069 | 40 | 40 |
| A | hsa-miR-184-4373113 | 35.894 | 40 | A | hsa-miR-200b-4395362 | 33.9 | 40 |
| A | hsa-miR-200c-4395411 | 32.978 | 32.921 | A | hsa-miR-220c-4395322 | 40 | 40 |
| A | hsa-miR-202-4395474 | 40 | 40 | A | hsa-miR-221-4373077 | 40 | 36.004 |
| A | hsa-miR-203-4373095 | 33.931 | 32.97 | A | hsa-miR-222-4395387 | 28.937 | 28.921 |
| A | hsa-miR-204-4373094 | 40 | 40 | A | hsa-miR-223-4395406 | 22.935 | 24.957 |
| A | hsa-miR-205-4373093 | 40 | 40 | A | hsa-miR-22-4373079 | 40 | 40 |
| A | hsa-miR-208-4373091 | 40 | 40 | A | hsa-miR-224-4395210 | 36.12 | 40 |
| A | hsa-miR-208b-4395401 | 40 | 40 | A | hsa-miR-23a-4373074 | 40 | 40 |
| A | hsa-miR-20a-4373286 | 26.999 | 28.974 | A | hsa-miR-23b-4373073 | 40 | 40 |
| A | hsa-miR-20b-4373263 | 29.971 | 31.966 | A | hsa-miR-24-4373072 | 26.968 | 26.926 |
| A | hsa-miR-210-4373089 | 40 | 40 | A | hsa-miR-25-4373071 | 30.963 | 30.954 |
| A | hsa-miR-211-4373088 | 33.99 | 40 | A | hsa-miR-26a-4395166 | 29.976 | 30.979 |
| A | hsa-miR-212-4373087 | 33.903 | 40 | A | hsa-miR-26b-4395167 | 30.938 | 32.967 |
| A | hsa-miR-21-4373090 | 29.957 | 31.011 | A | hsa-miR-27a-4373287 | 34.941 | 36.043 |
| A | hsa-miR-214-4395417 | 40 | 40 | A | hsa-miR-27b-4373068 | 40 | 40 |
| A | hsa-miR-215-4373084 | 32.045 | 33.993 | A | hsa-miR-28-3p-4395557 | 31.95 | 33.964 |
| A | hsa-miR-216a-4395331 | 40 | 40 | A | hsa-miR-28-5p-4373067 | 34.947 | 35.938 |
| A | hsa-miR-216b-4395437 | 40 | 40 | A | hsa-miR-296-3p-4395212 | 40 | 40 |
| A | hsa-miR-217-4395448 | 40 | 40 | A | hsa-miR-296-5p-4373066 | 40 | 35.979 |
| A | hsa-miR-218-4373081 | 40 | 40 | A | hsa-miR-298-4395301 | 40 | 40 |
| A | hsa-miR-219-1-3p-4395206 | 40 | 40 | A | hsa-miR-299-3p-4373189 | 40 | 40 |
| A | hsa-miR-219-2-3p-4395501 | 40 | 40 | A | hsa-miR-299-5p-4373188 | 40 | 40 |
| A | hsa-miR-219-5p-4373080 | 40 | 40 | A | hsa-miR-29a-4395223 | 33.059 | 31.979 |
| A | hsa-miR-220-4373078 | 40 | 40 | A | hsa-miR-29b-4373288 | 40 | 40 |
| A | hsa-miR-220b-4395317 | 40 | 40 | A | hsa-miR-29c-4395171 | 34.991 | 34.952 |
| A | hsa-miR-301a-4373064 | 32.928 | 32.946 | A | hsa-miR-339-3p-4395295 | 33.959 | 34.977 |
| A | hsa-miR-301b-4395503 | 40 | 40 | A | hsa-miR-339-5p-4395368 | 34.972 | 40 |
| A | hsa-miR-302a-4378070 | 40 | 40 | A | hsa-miR-33b-4395196 | 40 | 40 |
| A | hsa-miR-302b-4378071 | 40 | 40 | A | hsa-miR-340-4395369 | 34.992 | 35.999 |
| A | hsa-miR-302c-4378072 | 40 | 40 | A | hsa-miR-342-3p-4395371 | 28.977 | 30.008 |
| A | hsa-miR-30b-4373290 | 28.987 | 31.009 | A | hsa-miR-342-5p-4395258 | 40 | 40 |
| A | hsa-miR-30c-4373060 | 27.965 | 29.96 | A | hsa-miR-345-4395297 | 35.928 | 35.947 |
| A | hsa-miR-31-4395390 | 40 | 33.002 | A | hsa-miR-346-4373038 | 40 | 40 |
| A | hsa-miR-320-4395388 | 27.953 | 27.927 | A | hsa-miR-34a-4395168 | 40 | 40 |
| A | hsa-miR-323-3p-4395338 | 32.981 | 33.98 | A | hsa-miR-34c-5p-4373036 | 40 | 40 |
| A | hsa-miR-32-4395220 | 40 | 40 | A | hsa-miR-361-5p-4373035 | 35.98 | 40 |
| A | hsa-miR-324-3p-4395272 | 33.956 | 35.995 | A | hsa-miR-362-3p-4395228 | 40 | 40 |
| A | hsa-miR-324-5p-4373052 | 40 | 40 | A | hsa-miR-362-5p-4378092 | 36.011 | 40 |
| A | hsa-miR-325-4373051 | 40 | 40 | A | hsa-miR-363-4378090 | 40 | 40 |
| A | hsa-miR-326-4373050 | 40 | 40 | A | hsa-miR-365-4373194 | 32.947 | 33.961 |
| A | hsa-miR-328-4373049 | 30.966 | 32.957 | A | hsa-miR-367-4373034 | 40 | 40 |
| A | hsa-miR-329-4373191 | 37.307 | 40 | A | hsa-miR-369-3p-4373032 | 40 | 40 |
| A | hsa-miR-330-3p-4373047 | 35.071 | 34.992 | A | hsa-miR-369-5p-4373195 | 40 | 40 |
| A | hsa-miR-330-5p-4395341 | 40 | 40 | A | hsa-miR-370-4395386 | 40 | 40 |
| A | hsa-miR-331-3p-4373046 | 30.987 | 33.997 | A | hsa-miR-371-3p-4395235 | 40 | 40 |
| A | hsa-miR-331-5p-4395344 | 40 | 40 | A | hsa-miR-372-4373029 | 40 | 40 |
| A | hsa-miR-335-4373045 | 31.956 | 31.922 | A | hsa-miR-373-4378073 | 40 | 40 |
| A | hsa-miR-337-5p-4395267 | 40 | 40 | A | hsa-miR-374a-4373028 | 32.961 | 33.954 |
| A | hsa-miR-338-3p-4395363 | 40 | 35.966 | A | hsa-miR-374b-4381045 | 30.972 | 33.947 |
| A | hsa-miR-375-4373027 | 32.983 | 32.994 | A | hsa-miR-449b-4381011 | 40 | 40 |
| A | hsa-miR-376a-4373026 | 34.974 | 35.933 | A | hsa-miR-450a-4395414 | 40 | 40 |
| A | hsa-miR-376b-4373196 | 40 | 40 | A | hsa-miR-450b-3p-4395319 | 40 | 40 |
| A | hsa-miR-376c-4395233 | 32.96 | 32.957 | A | hsa-miR-450b-5p-4395318 | 40 | 40 |
| A | hsa-miR-377-4373025 | 40 | 40 | A | hsa-miR-451-4373360 | 27.939 | 28.974 |
| A | hsa-miR-379-4373349 | 40 | 40 | A | hsa-miR-452-4395440 | 40 | 40 |
| A | hsa-miR-380-4373022 | 40 | 40 | A | hsa-miR-453-4395429 | 40 | 40 |
| A | hsa-miR-381-4373020 | 35.983 | 40 | A | hsa-miR-454-4395434 | 33.932 | 33.949 |
| A | hsa-miR-382-4373019 | 40 | 40 | A | hsa-miR-455-3p-4395355 | 40 | 40 |
| A | hsa-miR-383-4373018 | 40 | 40 | A | hsa-miR-455-5p-4378098 | 40 | 40 |
| A | hsa-miR-384-4373017 | 40 | 40 | A | hsa-miR-483-5p-4395449 | 28.94 | 29.948 |
| A | hsa-miR-409-5p-4395442 | 40 | 40 | A | hsa-miR-484-4381032 | 26.957 | 27.959 |
| A | hsa-miR-410-4378093 | 35.974 | 35.993 | A | hsa-miR-485-3p-4378095 | 35.992 | 40 |
| A | hsa-miR-411-4381013 | 40 | 40 | A | hsa-miR-485-5p-4373212 | 40 | 40 |
| A | hsa-miR-412-4373199 | 40 | 40 | A | hsa-miR-486-3p-4395204 | 40 | 40 |
| A | hsa-miR-422a-4395408 | 40 | 40 | A | hsa-miR-486-5p-4378096 | 28.975 | 30.988 |
| A | hsa-miR-423-5p-4395451 | 30.975 | 31.987 | A | hsa-miR-487a-4378097 | 35.905 | 40 |
| A | hsa-miR-424-4373201 | 40 | 40 | A | hsa-miR-487b-4378102 | 35.961 | 40 |
| A | hsa-miR-425-4380926 | 28.913 | 29.863 | A | hsa-miR-488-4395468 | 40 | 40 |
| A | hsa-miR-429-4373203 | 40 | 40 | A | hsa-miR-489-4395469 | 35.972 | 35.955 |
| A | hsa-miR-431-4395173 | 40 | 40 | A | hsa-miR-490-3p-4373215 | 40 | 40 |
| A | hsa-miR-433-4373205 | 40 | 35.983 | A | hsa-miR-491-3p-4395471 | 40 | 40 |
| A | hsa-miR-448-4373206 | 40 | 40 | A | hsa-miR-491-5p-4381053 | 40 | 36.001 |
| A | hsa-miR-449a-4373207 | 40 | 40 | A | hsa-miR-492-4373217 | 40 | 40 |
| A | hsa-miR-493-4395475 | 40 | 40 | A | hsa-miR-513-5p-4395201 | 40 | 40 |
| A | hsa-miR-494-4395476 | 40 | 40 | A | hsa-miR-515-3p-4395480 | 40 | 40 |
| A | hsa-miR-495-4381078 | 33.047 | 40 | A | hsa-miR-515-5p-4373242 | 40 | 40 |
| A | hsa-miR-496-4386771 | 40 | 40 | A | hsa-miR-516a-5p-4395527 | 40 | 40 |
| A | hsa-miR-499-3p-4395538 | 40 | 40 | A | hsa-miR-516b-4395172 | 40 | 35.966 |
| A | hsa-miR-499-5p-4381047 | 40 | 40 | A | hsa-miR-517a-4395513 | 35.992 | 35.011 |
| A | hsa-miR-500-4395539 | 40 | 40 | A | hsa-miR-517b-4373244 | 40 | 40 |
| A | hsa-miR-501-3p-4395546 | 40 | 40 | A | hsa-miR-517c-4373264 | 35.87 | 36 |
| A | hsa-miR-501-5p-4373226 | 40 | 40 | A | hsa-miR-518a-3p-4395508 | 40 | 40 |
| A | hsa-miR-502-3p-4395194 | 35.941 | 40 | A | hsa-miR-518a-5p-4395507 | 40 | 40 |
| A | hsa-miR-502-5p-4373227 | 40 | 40 | A | hsa-miR-518b-4373246 | 35.961 | 36.004 |
| A | hsa-miR-503-4373228 | 40 | 40 | A | hsa-miR-518c-4395512 | 40 | 40 |
| A | hsa-miR-504-4395195 | 40 | 40 | A | hsa-miR-518d-3p-4373248 | 32.994 | 32.027 |
| A | hsa-miR-505-4395200 | 40 | 40 | A | hsa-miR-518d-5p-4395500 | 40 | 40 |
| A | hsa-miR-506-4373231 | 40 | 40 | A | hsa-miR-518e-4395506 | 40 | 40 |
| A | hsa-miR-507-4373232 | 40 | 40 | A | hsa-miR-518f-4395499 | 40 | 33.921 |
| A | hsa-miR-508-3p-4373233 | 40 | 40 | A | hsa-miR-519a-4395526 | 40 | 35.963 |
| A | hsa-miR-508-5p-4395203 | 40 | 40 | A | hsa-miR-519c-3p-4373251 | 35.948 | 40 |
| A | hsa-miR-509-3-5p-4395266 | 40 | 40 | A | hsa-miR-519d-4395514 | 40 | 40 |
| A | hsa-miR-509-5p-4395346 | 40 | 40 | A | hsa-miR-519e-4395481 | 40 | 40 |
| A | hsa-miR-510-4395352 | 40 | 40 | A | hsa-miR-520a-3p-4373268 | 40 | 40 |
| A | hsa-miR-511-4373236 | 35.962 | 40 | A | hsa-miR-520a-5p-4378085 | 36.001 | 40 |
| A | hsa-miR-512-3p-4381034 | 33.023 | 32.968 | A | hsa-miR-520b-4373252 | 40 | 40 |
| A | hsa-miR-512-5p-4373238 | 40 | 40 | A | hsa-miR-520d-5p-4395504 | 40 | 40 |
| A | hsa-miR-520e-4373255 | 40 | 40 | A | hsa-miR-548d-3p-4381008 | 40 | 40 |
| A | hsa-miR-520f-4373256 | 40 | 40 | A | hsa-miR-548d-5p-4395348 | 40 | 40 |
| A | hsa-miR-520g-4373257 | 40 | 40 | A | hsa-miR-551b-4380945 | 40 | 40 |
| A | hsa-miR-521-4373259 | 40 | 40 | A | hsa-miR-556-3p-4395456 | 40 | 40 |
| A | hsa-miR-522-4395524 | 35.073 | 40 | A | hsa-miR-556-5p-4395455 | 40 | 40 |
| A | hsa-miR-523-4395497 | 40 | 40 | A | hsa-miR-561-4380938 | 40 | 40 |
| A | hsa-miR-524-5p-4395174 | 40 | 40 | A | hsa-miR-570-4395458 | 40 | 40 |
| A | hsa-miR-525-3p-4395496 | 40 | 36.015 | A | hsa-miR-574-3p-4395460 | 30 | 30.987 |
| A | hsa-miR-525-5p-4378088 | 40 | 40 | A | hsa-miR-576-3p-4395462 | 40 | 40 |
| A | hsa-miR-526b-4395493 | 40 | 40 | A | hsa-miR-576-5p-4395461 | 40 | 40 |
| A | hsa-miR-532-3p-4395466 | 33.975 | 36.933 | A | hsa-miR-579-4395509 | 40 | 40 |
| A | hsa-miR-532-5p-4380928 | 35.019 | 36.024 | A | hsa-miR-582-3p-4395510 | 40 | 40 |
| A | hsa-miR-539-4378103 | 40 | 35.964 | A | hsa-miR-582-5p-4395175 | 40 | 40 |
| A | hsa-miR-541-4395312 | 40 | 40 | A | hsa-miR-589-4395520 | 40 | 40 |
| A | hsa-miR-542-3p-4378101 | 40 | 40 | A | hsa-miR-590-5p-4395176 | 34.948 | 34.948 |
| A | hsa-miR-542-5p-4395351 | 40 | 40 | A | hsa-miR-597-4380960 | 40 | 40 |
| A | hsa-miR-544-4395376 | 40 | 40 | A | hsa-miR-598-4395179 | 40 | 40 |
| A | hsa-miR-545-4395378 | 40 | 35.948 | A | hsa-miR-615-3p-4386777 | 40 | 40 |
| A | hsa-miR-548a-3p-4380948 | 33.93 | 33.941 | A | hsa-miR-615-5p-4395464 | 40 | 40 |
| A | hsa-miR-548a-5p-4395523 | 40 | 40 | A | hsa-miR-616-4395525 | 40 | 40 |
| A | hsa-miR-548b-3p-4380951 | 40 | 40 | A | hsa-miR-618-4380996 | 40 | 40 |
| A | hsa-miR-548b-5p-4395519 | 34.949 | 40 | A | hsa-miR-624-4395541 | 40 | 40 |
| A | hsa-miR-548c-3p-4380993 | 40 | 35.93 | A | hsa-miR-625-4395542 | 40 | 40 |
| A | hsa-miR-548c-5p-4395540 | 40 | 40 | A | hsa-miR-627-4380967 | 40 | 40 |
| A | hsa-miR-628-5p-4395544 | 34.91 | 40 | A | hsa-miR-885-3p-4395483 | 40 | 40 |
| A | hsa-miR-629-4395547 | 40 | 35.934 | A | hsa-miR-885-5p-4395407 | 29.955 | 30.958 |
| A | hsa-miR-636-4395199 | 32.992 | 40 | A | hsa-miR-886-3p-4395305 | 40 | 40 |
| A | hsa-miR-642-4380995 | 40 | 36.947 | A | hsa-miR-886-5p-4395304 | 36.029 | 40 |
| A | hsa-miR-651-4381007 | 40 | 40 | A | hsa-miR-887-4395485 | 40 | 40 |
| A | hsa-miR-652-4395463 | 35.993 | 35.992 | A | hsa-miR-888-4395323 | 35.958 | 40 |
| A | hsa-miR-653-4395403 | 40 | 40 | A | hsa-miR-889-4395313 | 40 | 40 |
| A | hsa-miR-654-3p-4395350 | 40 | 40 | A | hsa-miR-890-4395320 | 40 | 40 |
| A | hsa-miR-654-5p-4381014 | 40 | 40 | A | hsa-miR-891a-4395302 | 40 | 40 |
| A | hsa-miR-655-4381015 | 40 | 40 | A | hsa-miR-891b-4395321 | 40 | 40 |
| A | hsa-miR-660-4380925 | 40 | 35.933 | A | hsa-miR-892a-4395306 | 40 | 40 |
| A | hsa-miR-671-3p-4395433 | 33.99 | 40 | A | hsa-miR-92a-4395169 | 26.931 | 28.996 |
| A | hsa-miR-672-4395438 | 40 | 40 | A | hsa-miR-93-4373302 | 29.967 | 30.938 |
| A | hsa-miR-674-4395193 | 40 | 40 | A | hsa-miR-9-4373285 | 40 | 40 |
| A | hsa-miR-708-4395452 | 33.968 | 34.936 | A | hsa-miR-95-4373011 | 40 | 34.919 |
| A | hsa-miR-744-4395435 | 33.96 | 40 | A | hsa-miR-96-4373372 | 40 | 40 |
| A | hsa-miR-758-4395180 | 40 | 40 | A | hsa-miR-98-4373009 | 40 | 40 |
| A | hsa-miR-871-4395465 | 40 | 40 | A | hsa-miR-99a-4373008 | 34.991 | 33.998 |
| A | hsa-miR-872-4395375 | 40 | 40 | A | hsa-miR-99b-4373007 | 32.954 | 32.983 |
| A | hsa-miR-873-4395467 | 40 | 40 | B | hsa-let-7a*-4395418 | 40 | 40 |
| A | hsa-miR-874-4395379 | 40 | 40 | B | hsa-let-7b*-4395515 | 40 | 40 |
| A | hsa-miR-875-3p-4395315 | 40 | 40 | B | hsa-let-7d*-4378108 | 40 | 40 |
| A | hsa-miR-876-3p-4395336 | 40 | 40 | B | hsa-let-7e*-4395518 | 40 | 40 |
| A | hsa-miR-876-5p-4395316 | 40 | 40 | B | hsa-let-7f-1*-4395528 | 40 | 40 |
| B | hsa-let-7f-2*-4395529 | 36.96 | 40 | B | hsa-miR-143*-4395257 | 40 | 40 |
| B | hsa-let-7g*-4395229 | 40 | 40 | B | hsa-miR-144*-4395259 | 31.97 | 33.992 |
| B | hsa-let-7i*-4395283 | 40 | 40 | B | hsa-miR-145*-4395260 | 35.839 | 34.982 |
| B | hsa-miR-100*-4395253 | 40 | 40 | B | hsa-miR-146a*-4395274 | 40 | 40 |
| B | hsa-miR-101*-4395254 | 40 | 40 | B | hsa-miR-148a*-4395245 | 40 | 40 |
| B | hsa-miR-105*-4395279 | 40 | 40 | B | hsa-miR-148b*-4395271 | 40 | 40 |
| B | hsa-miR-106a*-4395281 | 40 | 40 | B | hsa-miR-149*-4395275 | 40 | 40 |
| B | hsa-miR-106b*-4395491 | 40 | 40 | B | hsa-miR-151-3p-4395365 | 28.932 | 28.963 |
| B | hsa-miR-10a*-4395399 | 40 | 40 | B | hsa-miR-154*-4378065 | 40 | 40 |
| B | hsa-miR-10b*-4395426 | 31.935 | 32.959 | B | hsa-miR-155*-4395398 | 40 | 40 |
| B | hsa-miR-122*-4395241 | 40 | 40 | B | hsa-miR-15a*-4395530 | 40 | 40 |
| B | hsa-miR-124*-4395308 | 40 | 40 | B | hsa-miR-15b*-4395284 | 40 | 40 |
| B | hsa-miR-125b-1*-4395489 | 40 | 40 | B | hsa-miR-16-1*-4395531 | 40 | 40 |
| B | hsa-miR-125b-2*-4395269 | 40 | 40 | B | hsa-miR-16-2*-4395282 | 40 | 40 |
| B | hsa-miR-126*-4373269 | 28.953 | 29.962 | B | hsa-miR-17*-4395532 | 40 | 40 |
| B | hsa-miR-130a*-4395242 | 40 | 40 | B | hsa-miR-181a*-4373086 | 40 | 40 |
| B | hsa-miR-130b*-4395225 | 40 | 40 | B | hsa-miR-181a-2*-4395428 | 40 | 34.965 |
| B | hsa-miR-132*-4395243 | 40 | 40 | B | hsa-miR-181c*-4395444 | 40 | 40 |
| B | hsa-miR-135a*-4395343 | 25.93 | 25.99 | B | hsa-miR-182*-4378066 | 40 | 40 |
| B | hsa-miR-135b*-4395270 | 40 | 40 | B | hsa-miR-183*-4395381 | 40 | 40 |
| B | hsa-miR-136*-4395211 | 40 | 40 | B | hsa-miR-185*-4395215 | 40 | 40 |
| B | hsa-miR-138-1*-4395273 | 28.994 | 29.072 | B | hsa-miR-186*-4395216 | 40 | 40 |
| B | hsa-miR-138-2*-4395255 | 40 | 40 | B | hsa-miR-188-5p-4395431 | 28.984 | 29.021 |
| B | hsa-miR-141*-4395256 | 40 | 40 | B | hsa-miR-18a*-4395534 | 40 | 40 |
| B | hsa-miR-18b*-4395421 | 40 | 40 | B | hsa-miR-23b*-4395237 | 40 | 40 |
| B | hsa-miR-190b-4395374 | 40 | 40 | B | hsa-miR-24-1*-4395551 | 40 | 40 |
| B | hsa-miR-192*-4395383 | 40 | 40 | B | hsa-miR-25*-4395553 | 40 | 40 |
| B | hsa-miR-193b*-4395477 | 40 | 40 | B | hsa-miR-26a-1*-4395554 | 40 | 40 |
| B | hsa-miR-194*-4395490 | 40 | 40 | B | hsa-miR-26a-2*-4395226 | 40 | 40 |
| B | hsa-miR-195*-4395218 | 40 | 40 | B | hsa-miR-26b*-4395555 | 40 | 35.962 |
| B | hsa-miR-19a*-4395535 | 40 | 40 | B | hsa-miR-27a*-4395556 | 35.99 | 34.985 |
| B | hsa-miR-19b-1*-4395536 | 35.918 | 36.052 | B | hsa-miR-27b*-4395285 | 40 | 40 |
| B | hsa-miR-19b-2*-4395537 | 40 | 40 | B | hsa-miR-29a*-4395558 | 40 | 40 |
| B | hsa-miR-200a*-4373273 | 40 | 40 | B | hsa-miR-29b-1*-4395276 | 40 | 40 |
| B | hsa-miR-200b*-4395385 | 40 | 40 | B | hsa-miR-29b-2*-4395277 | 40 | 40 |
| B | hsa-miR-200c*-4395397 | 40 | 40 | B | hsa-miR-29c*-4381131 | 40 | 36.97 |
| B | hsa-miR-202*-4395473 | 40 | 40 | B | hsa-miR-302a*-4395492 | 40 | 40 |
| B | hsa-miR-206-4373092 | 40 | 34.915 | B | hsa-miR-302b*-4395230 | 40 | 40 |
| B | hsa-miR-20a*-4395548 | 35.957 | 40 | B | hsa-miR-302c*-4373277 | 40 | 40 |
| B | hsa-miR-20b*-4395422 | 40 | 40 | B | hsa-miR-302d*-4395231 | 40 | 40 |
| B | hsa-miR-21*-4395549 | 40 | 40 | B | hsa-miR-302d-4373063 | 40 | 34.935 |
| B | hsa-miR-214*-4395404 | 40 | 40 | B | hsa-miR-30a*-4373062 | 32.903 | 33.995 |
| B | hsa-miR-218-2*-4395405 | 40 | 40 | B | hsa-miR-30a-4373061 | 28.933 | 28.918 |
| B | hsa-miR-22*-4395412 | 32.953 | 40 | B | hsa-miR-30b*-4395240 | 40 | 40 |
| B | hsa-miR-221*-4395207 | 40 | 40 | B | hsa-miR-30c-1*-4395219 | 40 | 40 |
| B | hsa-miR-222*-4395208 | 40 | 40 | B | hsa-miR-30c-2*-4395221 | 40 | 40 |
| B | hsa-miR-223*-4395209 | 33.017 | 32.988 | B | hsa-miR-30d*-4395416 | 40 | 40 |
| B | hsa-miR-23a*-4395550 | 40 | 40 | B | hsa-miR-30d-4373059 | 30.874 | 30.914 |
| B | hsa-miR-30e*-4373057 | 32.97 | 32.976 | B | hsa-miR-425*-4395413 | 33.987 | 33.952 |
| B | hsa-miR-30e-4395334 | 31.015 | 32.005 | B | hsa-miR-431*-4395423 | 40 | 40 |
| B | hsa-miR-32*-4395222 | 40 | 40 | B | hsa-miR-432*-4378076 | 40 | 40 |
| B | hsa-miR-335*-4395296 | 40 | 40 | B | hsa-miR-432-4373280 | 36.036 | 40 |
| B | hsa-miR-337-3p-4395268 | 40 | 40 | B | hsa-miR-452*-4395441 | 40 | 40 |
| B | hsa-miR-33a*-4395247 | 40 | 40 | B | hsa-miR-454*-4395185 | 40 | 40 |
| B | hsa-miR-340*-4395370 | 33.956 | 35.934 | B | hsa-miR-488*-4373213 | 40 | 40 |
| B | hsa-miR-34a*-4395427 | 35.976 | 40 | B | hsa-miR-493*-4373218 | 40 | 34.011 |
| B | hsa-miR-34b*-4373037 | 40 | 40 | B | hsa-miR-497*-4395479 | 40 | 40 |
| B | hsa-miR-361-3p-4395227 | 40 | 40 | B | hsa-miR-497-4373222 | 33.056 | 33.999 |
| B | hsa-miR-363*-4380917 | 40 | 40 | B | hsa-miR-498-4373223 | 40 | 40 |
| B | hsa-miR-367*-4395232 | 40 | 40 | B | hsa-miR-500*-4373225 | 40 | 40 |
| B | hsa-miR-373*-4373279 | 40 | 40 | B | hsa-miR-505*-4395198 | 33.989 | 35.012 |
| B | hsa-miR-374a*-4395236 | 40 | 40 | B | hsa-miR-509-3p-4395347 | 30.97 | 30.966 |
| B | hsa-miR-374b*-4395502 | 40 | 40 | B | hsa-miR-513-3p-4395202 | 34.018 | 40 |
| B | hsa-miR-376a*-4395238 | 40 | 40 | B | hsa-miR-516a-3p-4373183 | 34.978 | 36.955 |
| B | hsa-miR-377*-4395239 | 40 | 40 | B | hsa-miR-517*-4378078 | 40 | 40 |
| B | hsa-miR-378*-4373024 | 40 | 40 | B | hsa-miR-518c*-4378082 | 40 | 40 |
| B | hsa-miR-378-4395354 | 33.987 | 32.954 | B | hsa-miR-518e*-4395482 | 33.979 | 40 |
| B | hsa-miR-379*-4395244 | 40 | 40 | B | hsa-miR-518f*-4395498 | 40 | 40 |
| B | hsa-miR-380*-4373021 | 34.936 | 40 | B | hsa-miR-519b-3p-4395495 | 40 | 40 |
| B | hsa-miR-409-3p-4395443 | 30.945 | 30.965 | B | hsa-miR-519e*-4378084 | 40 | 40 |
| B | hsa-miR-411*-4395349 | 40 | 32.948 | B | hsa-miR-520c-3p-4395511 | 35.023 | 40 |
| B | hsa-miR-424*-4395420 | 40 | 40 | B | hsa-miR-520h-4373258 | 40 | 40 |
| B | hsa-miR-524-3p-4378087 | 40 | 35.976 | B | hsa-miR-571-4381016 | 31 | 40 |
| B | hsa-miR-526b*-4395494 | 33.952 | 35.944 | B | hsa-miR-572-4381017 | 32.909 | 33.96 |
| B | hsa-miR-541*-4395311 | 40 | 40 | B | hsa-miR-573-4381018 | 40 | 40 |
| B | hsa-miR-543-4395487 | 40 | 34.05 | B | hsa-miR-575-4381020 | 40 | 40 |
| B | hsa-miR-545*-4395377 | 40 | 40 | B | hsa-miR-578-4381022 | 40 | 40 |
| B | hsa-miR-549-4380921 | 40 | 40 | B | hsa-miR-580-4381024 | 40 | 40 |
| B | hsa-miR-550*-4380954 | 40 | 40 | B | hsa-miR-581-4386744 | 40 | 40 |
| B | hsa-miR-550-4395521 | 40 | 40 | B | hsa-miR-583-4381025 | 40 | 40 |
| B | hsa-miR-551a-4380929 | 40 | 40 | B | hsa-miR-584-4381026 | 40 | 32.01 |
| B | hsa-miR-551b*-4395457 | 40 | 40 | B | hsa-miR-585-4381027 | 40 | 40 |
| B | hsa-miR-552-4380930 | 40 | 40 | B | hsa-miR-586-4380949 | 40 | 40 |
| B | hsa-miR-553-4380931 | 40 | 40 | B | hsa-miR-587-4380950 | 40 | 40 |
| B | hsa-miR-554-4380932 | 40 | 40 | B | hsa-miR-588-4380952 | 40 | 40 |
| B | hsa-miR-555-4380933 | 35.99 | 40 | B | hsa-miR-589*-4380953 | 40 | 40 |
| B | hsa-miR-557-4380935 | 40 | 40 | B | hsa-miR-591-4380955 | 34.497 | 35.385 |
| B | hsa-miR-558-4380936 | 40 | 40 | B | hsa-miR-592-4380956 | 40 | 40 |
| B | hsa-miR-559-4380937 | 40 | 40 | B | hsa-miR-593*-4380957 | 40 | 40 |
| B | hsa-miR-562-4380939 | 40 | 40 | B | hsa-miR-593-4395522 | 40 | 40 |
| B | hsa-miR-563-4380940 | 40 | 40 | B | hsa-miR-595-4395178 | 40 | 40 |
| B | hsa-miR-564-4380941 | 33.952 | 40 | B | hsa-miR-596-4380959 | 40 | 40 |
| B | hsa-miR-565-4380942 | 40 | 40 | B | hsa-miR-599-4380962 | 40 | 40 |
| B | hsa-miR-566-4380943 | 40 | 40 | B | hsa-miR-600-4380963 | 40 | 40 |
| B | hsa-miR-567-4380944 | 40 | 40 | B | hsa-miR-601-4380965 | 40 | 40 |
| B | hsa-miR-569-4380946 | 40 | 40 | B | hsa-miR-603-4380972 | 40 | 40 |
| B | hsa-miR-604-4380973 | 40 | 35.941 | B | hsa-miR-633-4380979 | 40 | 40 |
| B | hsa-miR-605-4386742 | 31.444 | 35.108 | B | hsa-miR-634-4380981 | 40 | 40 |
| B | hsa-miR-606-4380974 | 40 | 40 | B | hsa-miR-635-4380982 | 40 | 36.932 |
| B | hsa-miR-607-4380975 | 40 | 40 | B | hsa-miR-637-4380985 | 40 | 40 |
| B | hsa-miR-608-4380976 | 40 | 40 | B | hsa-miR-638-4380986 | 35.941 | 40 |
| B | hsa-miR-609-4380978 | 40 | 40 | B | hsa-miR-639-4380987 | 33.093 | 40 |
| B | hsa-miR-610-4380980 | 30.943 | 32.009 | B | hsa-miR-640-4386743 | 33.972 | 40 |
| B | hsa-miR-612-4380983 | 40 | 40 | B | hsa-miR-641-4380988 | 40 | 40 |
| B | hsa-miR-613-4380989 | 40 | 40 | B | hsa-miR-643-4380997 | 40 | 40 |
| B | hsa-miR-614-4380990 | 40 | 40 | B | hsa-miR-644-4380999 | 33.973 | 40 |
| B | hsa-miR-616*-4380992 | 40 | 40 | B | hsa-miR-645-4381000 | 35.204 | 35.956 |
| B | hsa-miR-617-4380994 | 40 | 40 | B | hsa-miR-646-4381002 | 40 | 40 |
| B | hsa-miR-619-4380998 | 40 | 40 | B | hsa-miR-647-4381003 | 40 | 40 |
| B | hsa-miR-621-4381001 | 40 | 40 | B | hsa-miR-648-4381004 | 40 | 40 |
| B | hsa-miR-622-4380961 | 40 | 40 | B | hsa-miR-649-4381005 | 35.021 | 40 |
| B | hsa-miR-623-4386740 | 40 | 40 | B | hsa-miR-650-4381006 | 31.965 | 40 |
| B | hsa-miR-624*-4380964 | 40 | 40 | B | hsa-miR-656-4380920 | 40 | 40 |
| B | hsa-miR-625*-4395543 | 30.96 | 32.015 | B | hsa-miR-657-4380922 | 40 | 40 |
| B | hsa-miR-626-4380966 | 40 | 40 | B | hsa-miR-658-4380923 | 40 | 40 |
| B | hsa-miR-628-3p-4395545 | 33.51 | 35.006 | B | hsa-miR-659-4380924 | 40 | 40 |
| B | hsa-miR-629*-4380969 | 34.976 | 36.023 | B | hsa-miR-661-4381009 | 30.032 | 40 |
| B | hsa-miR-630-4380970 | 31.007 | 30.998 | B | hsa-miR-662-4381010 | 40 | 40 |
| B | hsa-miR-631-4380971 | 40 | 40 | B | hsa-miR-668-4395181 | 40 | 40 |
| B | hsa-miR-632-4380977 | 32.982 | 31.945 | B | hsa-miR-675-4395192 | 40 | 40 |
| B | hsa-miR-708*-4395453 | 40 | 40 | B | hsa-miR-92a-1*-4395248 | 40 | 35.962 |
| B | hsa-miR-7-1*-4381118 | 31.988 | 33.008 | B | hsa-miR-92a-2*-4395249 | 40 | 40 |
| B | hsa-miR-7-2*-4395425 | 40 | 40 | B | hsa-miR-92b*-4395454 | 40 | 40 |
| B | hsa-miR-7-4378130 | 40 | 40 | B | hsa-miR-93*-4395250 | 40 | 35.96 |
| B | hsa-miR-744*-4395436 | 35.039 | 40 | B | hsa-miR-933-4395287 | 40 | 40 |
| B | hsa-miR-760-4395439 | 29.952 | 29.981 | B | hsa-miR-934-4395288 | 40 | 40 |
| B | hsa-miR-766-4395177 | 28.071 | 29.919 | B | hsa-miR-935-4395289 | 40 | 40 |
| B | hsa-miR-767-3p-4395184 | 40 | 40 | B | hsa-miR-936-4395290 | 40 | 40 |
| B | hsa-miR-767-5p-4395182 | 40 | 40 | B | hsa-miR-937-4395291 | 40 | 40 |
| B | hsa-miR-768-3p-4395188 | 40 | 36.004 | B | hsa-miR-938-4395292 | 40 | 40 |
| B | hsa-miR-769-3p-4395190 | 40 | 40 | B | hsa-miR-939-4395293 | 40 | 40 |
| B | hsa-miR-769-5p-4395186 | 31.495 | 31.967 | B | hsa-miR-941-4395294 | 40 | 40 |
| B | hsa-miR-770-5p-4395189 | 40 | 40 | B | hsa-miR-942-4395298 | 33.972 | 35.947 |
| B | hsa-miR-801-4395183 | 35.444 | 34.254 | B | hsa-miR-943-4395299 | 40 | 40 |
| B | hsa-miR-875-5p-4395314 | 40 | 40 | B | hsa-miR-944-4395300 | 40 | 40 |
| B | hsa-miR-877-4395402 | 29.974 | 29.954 | B | hsa-miR-96*-4395251 | 40 | 40 |
| B | hsa-miR-888*-4395324 | 40 | 40 | B | hsa-miR-99a*-4395252 | 40 | 40 |
| B | hsa-miR-892b-4395325 | 40 | 40 | B | hsa-miR-99b*-4395307 | 32.992 | 32.968 |
| B | hsa-miR-9*-4395342 | 33.939 | 40 | B | hsa-miR-922-4395263 | 40 | 40 |
| B | hsa-miR-920-4395261 | 40 | 40 | B | hsa-miR-923-4395264 | 28.958 | 27.817 |
| B | hsa-miR-921-4395262 | 40 | 40 | B | hsa-miR-924-4395265 | 40 | 40 |
