# Supplementary material for: Early Second-Trimester Serum MiRNA Profiling Predicts Gestational Diabetes Mellitus
Source: PLoS One. 2011 Aug 24;6(8):e23925. doi: 10.1371/journal.pone.0023925 (PMC3161072; doi:10.1371/journal.pone.0023925)
Supplement: Table S2 — AB assay ID of the miRNAs. (DOC) [file pone.0023925.s002.doc]

**Table S2. AB assay ID of the miRNAs**

| miRNA | Assay type | AB assay ID |
| --- | --- | --- |
| hsa-miR-1 | Mature miRNA | [002222](https://products.appliedbiosystems.com:443/ab/en/US/adirect/ab?cmd=ABAssayDetailDisplay&assayID=002222&Fs=y&adv_phrase3=EXACT&adv_phrase2=EXACT&adv_phrase1=EXACT&assayType=mirna&catID=601803&SearchRequest.Common.SortSpec=ASSAY_NAME+asc&searchValue=null&searchBy=null&adv_kw_filter3=ALL&srchType=keyword&adv_kw_filter2=ALL&SearchRequest.Common.QueryText=hsa-miR-1&kwdropdown=all_microRNA&adv_kw_filter1=ALL&inventoried=*&adv_query_text3=&searchType=keyword&adv_query_text2=&adv_query_text1=&adv_boolean3=AND&displayAdvSearchResults=null&SearchRequest.Common.ResultsPerPage=25&adv_boolean2=AND&adv_boolean1=AND&chkBatchQueryText=false&kwfilter=ALL&SearchRequest.Common.PageNumber=1&isSL=null&msgType=ABmiRNAKeywordResults) |
| hsa-miR-125b | Mature miRNA | 000449 |
| hsa-miR-132 | Mature miRNA | [000457](https://products.appliedbiosystems.com:443/ab/en/US/adirect/ab?cmd=ABAssayDetailDisplay&assayID=000457&Fs=y&adv_phrase3=EXACT&adv_phrase2=EXACT&adv_phrase1=EXACT&assayType=mirna&catID=601803&SearchRequest.Common.SortSpec=ASSAY_NAME+asc&searchValue=null&searchBy=null&adv_kw_filter3=ALL&srchType=keyword&adv_kw_filter2=ALL&SearchRequest.Common.QueryText=hsa-miR-132&kwdropdown=all_microRNA&adv_kw_filter1=ALL&adv_query_text3=&searchType=keyword&adv_query_text2=&adv_query_text1=&adv_boolean3=AND&displayAdvSearchResults=null&adv_boolean2=AND&adv_boolean1=AND&chkBatchQueryText=false&kwfilter=ALL&SearchRequest.Common.PageNumber=1&isSL=null&msgType=ABmiRNAKeywordResults) |
| hsa-miR-29a | Mature miRNA | [002112](https://products.appliedbiosystems.com:443/ab/en/US/adirect/ab?cmd=ABAssayDetailDisplay&assayID=002112&Fs=y&adv_phrase3=EXACT&adv_phrase2=EXACT&adv_phrase1=EXACT&assayType=mirna&catID=601803&SearchRequest.Common.SortSpec=ASSAY_NAME+asc&searchValue=null&searchBy=null&adv_kw_filter3=ALL&srchType=keyword&adv_kw_filter2=ALL&SearchRequest.Common.QueryText=hsa-miR-29a&kwdropdown=all_microRNA&adv_kw_filter1=ALL&inventoried=*&adv_query_text3=&searchType=keyword&adv_query_text2=&adv_query_text1=&adv_boolean3=AND&displayAdvSearchResults=null&SearchRequest.Common.ResultsPerPage=25&adv_boolean2=AND&adv_boolean1=AND&chkBatchQueryText=false&kwfilter=ALL&SearchRequest.Common.PageNumber=1&isSL=null&msgType=ABmiRNAKeywordResults) |
| hsa-miR-203 | Mature miRNA | [000507](https://products.appliedbiosystems.com:443/ab/en/US/adirect/ab?cmd=ABAssayDetailDisplay&assayID=000507&Fs=y&adv_phrase3=EXACT&adv_phrase2=EXACT&adv_phrase1=EXACT&assayType=mirna&catID=601803&SearchRequest.Common.SortSpec=ASSAY_NAME+asc&searchValue=null&searchBy=null&adv_kw_filter3=ALL&srchType=keyword&adv_kw_filter2=ALL&SearchRequest.Common.QueryText=hsa-miR-203&kwdropdown=all_microRNA&adv_kw_filter1=ALL&inventoried=*&adv_query_text3=&searchType=keyword&adv_query_text2=&adv_query_text1=&adv_boolean3=AND&displayAdvSearchResults=null&SearchRequest.Common.ResultsPerPage=25&adv_boolean2=AND&adv_boolean1=AND&chkBatchQueryText=false&kwfilter=ALL&SearchRequest.Common.PageNumber=1&isSL=null&msgType=ABmiRNAKeywordResults) |
| hsa-miR-222 | Mature miRNA | [002276](https://products.appliedbiosystems.com:443/ab/en/US/adirect/ab?cmd=ABAssayDetailDisplay&assayID=002276&Fs=y&adv_phrase3=EXACT&adv_phrase2=EXACT&adv_phrase1=EXACT&assayType=mirna&catID=601803&SearchRequest.Common.SortSpec=ASSAY_NAME+asc&searchValue=null&searchBy=null&adv_kw_filter3=ALL&srchType=keyword&adv_kw_filter2=ALL&SearchRequest.Common.QueryText=hsa-miR-222&kwdropdown=all_microRNA&adv_kw_filter1=ALL&inventoried=*&adv_query_text3=&searchType=keyword&adv_query_text2=&adv_query_text1=&adv_boolean3=AND&displayAdvSearchResults=null&SearchRequest.Common.ResultsPerPage=25&adv_boolean2=AND&adv_boolean1=AND&chkBatchQueryText=false&kwfilter=ALL&SearchRequest.Common.PageNumber=1&isSL=null&msgType=ABmiRNAKeywordResults) |
| hsa-miR-378 | Mature miRNA | [002243](https://products.appliedbiosystems.com:443/ab/en/US/adirect/ab?cmd=ABAssayDetailDisplay&assayID=002243&Fs=y&adv_phrase3=EXACT&adv_phrase2=EXACT&adv_phrase1=EXACT&assayType=mirna&catID=601803&SearchRequest.Common.SortSpec=ASSAY_NAME+asc&searchValue=null&searchBy=null&adv_kw_filter3=ALL&srchType=keyword&adv_kw_filter2=ALL&SearchRequest.Common.QueryText=hsa-miR-378&kwdropdown=all_microRNA&adv_kw_filter1=ALL&inventoried=*&adv_query_text3=&searchType=keyword&adv_query_text2=&adv_query_text1=&adv_boolean3=AND&displayAdvSearchResults=null&SearchRequest.Common.ResultsPerPage=25&adv_boolean2=AND&adv_boolean1=AND&chkBatchQueryText=false&kwfilter=ALL&SearchRequest.Common.PageNumber=1&isSL=null&msgType=ABmiRNAKeywordResults) |
| hsa-miR-518d-3p | Mature miRNA | [001159](https://products.appliedbiosystems.com:443/ab/en/US/adirect/ab?cmd=ABAssayDetailDisplay&assayID=001159&Fs=y&adv_phrase3=EXACT&adv_phrase2=EXACT&adv_phrase1=EXACT&assayType=mirna&catID=601803&SearchRequest.Common.SortSpec=ASSAY_NAME+asc&searchValue=null&searchBy=null&adv_kw_filter3=ALL&srchType=keyword&adv_kw_filter2=ALL&SearchRequest.Common.QueryText=hsa-miR-518d&kwdropdown=all_microRNA&adv_kw_filter1=ALL&adv_query_text3=&searchType=keyword&adv_query_text2=&adv_query_text1=&adv_boolean3=AND&displayAdvSearchResults=null&adv_boolean2=AND&adv_boolean1=AND&chkBatchQueryText=false&kwfilter=ALL&SearchRequest.Common.PageNumber=1&isSL=null&msgType=ABmiRNAKeywordResults) |
| hsa-miR-632 | Mature miRNA | [001572](https://products.appliedbiosystems.com:443/ab/en/US/adirect/ab?cmd=ABAssayDetailDisplay&assayID=001572&Fs=y&adv_phrase3=EXACT&adv_phrase2=EXACT&adv_phrase1=EXACT&assayType=mirna&catID=601803&SearchRequest.Common.SortSpec=ASSAY_NAME+asc&searchValue=null&searchBy=null&adv_kw_filter3=ALL&srchType=keyword&adv_kw_filter2=ALL&SearchRequest.Common.QueryText=hsa-miR-632&kwdropdown=all_microRNA&adv_kw_filter1=ALL&inventoried=*&adv_query_text3=&searchType=keyword&adv_query_text2=&adv_query_text1=&adv_boolean3=AND&displayAdvSearchResults=null&SearchRequest.Common.ResultsPerPage=25&adv_boolean2=AND&adv_boolean1=AND&chkBatchQueryText=false&kwfilter=ALL&SearchRequest.Common.PageNumber=1&isSL=null&msgType=ABmiRNAKeywordResults) |
| hsa-miR-923 | Mature miRNA | [002153](https://products.appliedbiosystems.com:443/ab/en/US/adirect/ab?cmd=ABAssayDetailDisplay&assayID=002153&Fs=y&adv_phrase3=EXACT&adv_phrase2=EXACT&adv_phrase1=EXACT&assayType=mirna&catID=601803&SearchRequest.Common.SortSpec=ASSAY_NAME+asc&searchValue=null&searchBy=null&adv_kw_filter3=ALL&srchType=keyword&adv_kw_filter2=ALL&SearchRequest.Common.QueryText=hsa-miR-923&kwdropdown=all_microRNA&adv_kw_filter1=ALL&inventoried=*&adv_query_text3=&searchType=keyword&adv_query_text2=&adv_query_text1=&adv_boolean3=AND&displayAdvSearchResults=null&SearchRequest.Common.ResultsPerPage=25&adv_boolean2=AND&adv_boolean1=AND&chkBatchQueryText=false&kwfilter=ALL&SearchRequest.Common.PageNumber=1&isSL=null&msgType=ABmiRNAKeywordResults) |
| hsa-miR-99a | Mature miRNA | [000435](https://products.appliedbiosystems.com:443/ab/en/US/adirect/ab?cmd=ABAssayDetailDisplay&assayID=000435&Fs=y&adv_phrase3=EXACT&adv_phrase2=EXACT&adv_phrase1=EXACT&assayType=mirna&catID=601803&SearchRequest.Common.SortSpec=ASSAY_NAME+asc&searchValue=null&searchBy=null&adv_kw_filter3=ALL&srchType=keyword&adv_kw_filter2=ALL&SearchRequest.Common.QueryText=hsa-miR-99a&kwdropdown=all_microRNA&adv_kw_filter1=ALL&inventoried=*&adv_query_text3=&searchType=keyword&adv_query_text2=&adv_query_text1=&adv_boolean3=AND&displayAdvSearchResults=null&SearchRequest.Common.ResultsPerPage=25&adv_boolean2=AND&adv_boolean1=AND&chkBatchQueryText=false&kwfilter=ALL&SearchRequest.Common.PageNumber=1&isSL=null&msgType=ABmiRNAKeywordResults) |
| Cel-miR-39 | Control miRNA | [000200](https://products.appliedbiosystems.com:443/ab/en/US/adirect/ab?cmd=ABAssayDetailDisplay&assayID=000200&Fs=y&adv_phrase3=EXACT&adv_phrase2=EXACT&adv_phrase1=EXACT&assayType=mirna&catID=601803&SearchRequest.Common.SortSpec=ASSAY_NAME+asc&searchValue=null&searchBy=null&adv_kw_filter3=ALL&srchType=keyword&adv_kw_filter2=ALL&SearchRequest.Common.QueryText=cel-miR-39&kwdropdown=all_microRNA&adv_kw_filter1=ALL&inventoried=*&adv_query_text3=&searchType=keyword&adv_query_text2=&adv_query_text1=&adv_boolean3=AND&displayAdvSearchResults=null&SearchRequest.Common.ResultsPerPage=25&adv_boolean2=AND&adv_boolean1=AND&chkBatchQueryText=false&kwfilter=ALL&SearchRequest.Common.PageNumber=1&isSL=null&msgType=ABmiRNAKeywordResults) |
